# MGFNet: A Multi-Granularity Fusion Network with Coupling-Guided Sparse Routing for Hybrid EEG-fNIRS Decoding

**DOI:** 10.3390/s26113402

**Published:** 2026-05-27

**Authors:** Yan Zhang, Xiaoyu Gong, Xiaoyang Yuan

**Affiliations:** School of Electrical Engineering and Automation, Harbin Institute of Technology, Harbin 150001, China; 24s106272@stu.hit.edu.cn (X.G.); yuanxy@stu.hit.edu.cn (X.Y.)

**Keywords:** EEG-fNIRS hybrid BCI, multimodal fusion, CGSCR, adaptive routing, cross-modal coupling, robustness

## Abstract

Hybrid brain–computer interfaces (BCIs) have attracted growing research attention because they combine the millisecond-level temporal resolution of electroencephalography (EEG) with the spatially informative hemodynamic responses of functional near-infrared spectroscopy (fNIRS). However, most existing deep fusion methods rely on static late-fusion strategies, which tend to underexploit latent cross-modal dependencies and are vulnerable to modality-specific signal degradation. To address these limitations, we propose MGFNet, a multi-granularity fusion network for hybrid BCI decoding. MGFNet contains three components: (1) intra-modal encoders that learn modality-specific spatiotemporal representations from EEG, oxygenated hemoglobin (HbO), and deoxygenated hemoglobin (HbR) signals; (2) cross-modal interaction encoders that temporally align paired modalities and use dilated convolutions to capture long-range EEG-fNIRS dependencies; and (3) a Coupling-Guided Sparse Component Routing (CGSCR) module that estimates sample-specific cross-modal coupling and performs adaptive discrete routing. We further introduce a deep supervision strategy to stabilize optimization and improve branch-level discriminability. Under a within-subject held-out evaluation protocol on a public benchmark dataset, MGFNet achieved classification accuracies of 99.40% on the n-back task and 99.03% on the word generation (WG) task, outperforming representative comparison methods evaluated under a matched protocol. Ablation studies further confirmed the contributions of the intra-modal encoders, the cross-modal interaction encoders, and the CGSCR module. Under controlled EEG corruption with additive white Gaussian noise at −10 dB, MGFNet outperformed a static-fusion variant by 9.23 percentage points on the n-back task and 6.31 percentage points on the WG task. These results support the effectiveness of MGFNet in the present offline within-subject setting and indicate improved robustness under controlled single-modality degradation.

## 1. Introduction

Non-invasive brain–computer interfaces (BCIs) provide a direct pathway for human–machine interaction and play a crucial role in neurorehabilitation and cognitive state monitoring [1,2,3]. However, a single sensing modality is often insufficient to capture the complex spatiotemporal dynamics of brain activity required for high-performance BCI systems. Therefore, hybrid brain–computer interfaces (hBCIs) have been developed to overcome the inherent limitations of single-modality systems by integrating electrophysiological signals (EEG), which offer high temporal resolution, with hemodynamic signals (fNIRS), which provide high spatial resolution [4,5,6]. By integrating complementary information across different spatiotemporal scales, such multimodal fusion is expected to improve both decoding accuracy and the information transfer rate (ITR) of BCI systems in realistic application scenarios [7,8].

Depending on the stage of information integration, existing fusion strategies for EEG and fNIRS are primarily categorized into decision-level, feature-level, and data-level fusion [9,10]. Early studies predominantly relied on decision-level fusion. For example, when releasing a benchmark public dataset, Shin et al. [11] used Linear Discriminant Analysis (LDA) to classify EEG and fNIRS separately and combined the two predictions through weighted voting. However, this strategy treats the modalities as fully independent streams and neglects potential correlations in the joint feature space. To exploit complementary information, feature-level fusion gradually became the mainstream paradigm [12,13,14]. Traditional approaches typically concatenate handcrafted features directly [15], while classifier-design studies for EEG-fNIRS bimodal BCI have also shown that appropriate feature construction and classifier selection are important for reliable hybrid decoding [16]. However, such feature-level pipelines often suffer from the curse of dimensionality and make it difficult to effectively mine deep cross-modal nonlinear relationships [17].

Driven by recent advances in deep learning, end-to-end representation learning has gradually replaced traditional handcrafted-feature concatenation pipelines in physiological-signal decoding. Modern models can learn discriminative temporal, spatial, and connectivity patterns directly from raw or minimally processed inputs [18,19,20]. Recent high-quality studies have further highlighted this transition. For example, a recent review on EEG-based emotion recognition summarized how modern end-to-end deep architectures have improved representation learning, robustness, and generalization in EEG decoding tasks [21]. In addition, an EEG-based driver fatigue detection study demonstrated that integrating graph attention with mutual-information-driven connectivity can enhance end-to-end learning by explicitly modeling inter-channel relationships [22]. In this context, convolutional neural networks (CNNs) and long short-term memory (LSTM) networks have been widely adopted for automated feature extraction [23], while hybrid CNN-LSTM architectures have been used to jointly model spatial and temporal dependencies [24,25]. To further address long-range sequence modeling, recent studies have introduced attention mechanisms and Transformer-based architectures [26,27,28]. Models such as STFT-MDNF [29] and MBC-ATT [30] have also employed attention mechanisms [31,32] to model cross-modal correlations, achieving strong performance on standard hybrid BCI benchmarks. Correlation-aware network designs have also been investigated for hybrid EEG-fNIRS BCI decoding [33].

Despite their promising performance, most of these methods still rely on late fusion [34] or other static fusion strategies, which exhibit two main limitations in practice. First, many existing architectures do not explicitly model the deep statistical dependencies between heterogeneous signals. Instead, EEG and fNIRS are often processed as separate streams and combined only at the final stage through relatively simple weighting schemes. This segregated processing tends to overlook the nonlinear cross-modal correlations that arise in the latent feature space [35,36]. Second, these systems generally lack the ability to adapt dynamically to modality-specific noise. In real-world settings, both EEG and fNIRS are susceptible to signal contamination, such as electromyographic (EMG) artifacts in EEG and optode coupling instability in fNIRS [37,38,39]. However, static fusion and conventional soft-attention mechanisms typically assign continuous weights to all inputs while keeping the inference pathway fixed. As a result, they tend to reflect global dataset-level statistics rather than sample-specific fluctuations in signal quality. When the signal-to-noise ratio (SNR) of one modality deteriorates substantially, the resulting noise may propagate through the fusion stage and degrade the final decision boundary, thereby reducing the robustness of the system [40,41].

To handle non-stationary signals, dynamic neural networks, such as Mixture-of-Experts models and Gumbel-Softmax-based conditional computation [42,43,44], provide a paradigm that adaptively adjusts computational pathways according to the input [45]. However, the application of explicit adaptive routing mechanisms to non-stationary noise and modality heterogeneity in EEG-fNIRS multimodal BCIs is still at an early stage. Most existing works still assume that the complementary relationship between modalities remains stable across samples.

To bridge this gap, we propose MGFNet (Multi-Granularity Fusion Network), an architecture designed for the integrated decoding of heterogeneous EEG-fNIRS signals. Unlike traditional approaches that rely on hand-crafted priors, static fusion rules, or continuous soft weighting, MGFNet introduces a discrete gating strategy that searches the EEG-fNIRS feature subspace for sample-specific informative combinations. This design enables MGFNet to explicitly capture cross-modal interactions and to adaptively adjust feature routing according to latent cross-modal coupling [46,47], maintaining high robustness even when one modality is severely compromised. Beyond hybrid EEG-fNIRS BCI, multi-granularity feature enhancement has also been explored in other deep-learning research areas [48], indicating that granularity-aware representation design is a generally applicable principle for heterogeneous data modeling.

The main contributions of this work are summarized as follows:

- **We develop a multi-granularity feature interaction architecture for hybrid EEG-fNIRS decoding.** The proposed framework combines intra-modal representation learning with hierarchical cross-modal interaction modeling, enabling it to capture both modality-specific spatiotemporal patterns and complementary information across different granularities. In this way, MGFNet alleviates the limited interaction problem in conventional late-fusion schemes and improves the alignment and integration of heterogeneous physiological signals.
- **We propose a Coupling-Guided Sparse Component Routing (CGSCR) module for adaptive cross-modal fusion.** The proposed module introduces a data-driven cross-modal coupling mechanism to estimate latent statistical dependencies between EEG spectral representations and fNIRS hemodynamic representations. Based on these coupling cues, CGSCR performs discrete gating to adaptively route informative feature components rather than relying on fixed fusion weights. This design enables the model to form input-dependent cross-modal cooperation and to better tolerate single-modality degradation during fusion.
- **We demonstrate improved decoding performance and controlled-noise resilience on a public hybrid BCI benchmark.** Under the within-subject evaluation protocol on the public dataset of Shin et al. (2018) [49], MGFNet reached classification accuracies of 99.40% and 99.03% on the n-back and word generation (WG) tasks, respectively, exceeding representative comparison methods evaluated under a matched protocol. In addition, ablation studies verified the contributions of the intra-modal encoders, the cross-modal interaction encoders, and the CGSCR module. Under controlled EEG corruption with additive white Gaussian noise at −10 dB, MGFNet further outperformed its static-fusion variant by 9.23 and 6.31 percentage points on the n-back and WG tasks, respectively, indicating improved resilience under controlled single-modality degradation.

## 2. Dataset

### 2.1. Dataset Description

This study used the publicly available multimodal BCI dataset published by Shin et al. (2018) [49]. The dataset comprises synchronously recorded EEG and fNIRS signals from 26 healthy participants (mean age: 26.1 ± 3.5 years) during cognitive-task performance. This study focused on two tasks from the dataset:

**n-back task (working-memory load):** Participants were required to determine whether the currently displayed number matched the one presented *n* steps earlier [50]. The experiment included three difficulty levels: 0-back, 2-back, and 3-back, representing low, medium, and high cognitive load levels, respectively. Each task block consisted of a 2 s instruction period, a 40 s task period, and a 20 s rest period.

**Word generation (WG) task:** Participants were instructed either to perform word association based on an initial letter presented on the screen (WG condition) or to remain relaxed while fixating on a cross (Baseline condition). Each trial comprised a 2 s instruction period, a 10 s task period, and a 13–15 s rest period.

EEG was recorded with a BrainAmp amplifier (Brain Products GmbH, Gilching, Germany) at 1000 Hz from 30 channels, and fNIRS was recorded with a NIRScout system (NIRx Medizintechnik GmbH, Berlin, Germany) at 10.4 Hz from 36 channels.

### 2.2. Data Preprocessing and Sample Construction

To ensure a fair comparison with existing state-of-the-art (SOTA) methods, we strictly adhered to standard data preprocessing pipelines. **EEG Preprocessing:** The raw EEG data were downsampled to 200 Hz and filtered with a 1–40 Hz band-pass filter to reduce high-frequency noise and low-frequency drift while retaining theta, alpha, beta, and gamma band information. Electrooculogram (EOG) artifacts were removed using a standard correction algorithm. Recent attention-based deep-learning methods for EEG artifact removal have also been proposed [51]; in this study, we retained the standard preprocessing pipeline used in prior work on the same benchmark to maintain comparability. **fNIRS Preprocessing:** The raw optical intensity data were converted into concentration changes of oxygenated hemoglobin (HbO) and deoxygenated hemoglobin (HbR) according to the Modified Beer-Lambert Law (MBLL). The data were subsequently downsampled to 10 Hz and low-pass filtered to remove physiological noise, following the preprocessing conventions commonly adopted in fNIRS studies [52].

For data segmentation, we followed the sliding-window strategy used in the MBC-ATT study. For both the n-back and WG tasks, data were extracted from the task periods using a 5 s window with a 1 s step size. Following segmentation, the dimensions of each EEG sample were $X_{EEG}\in {\mathbb{R}}^{30\times 1000}$, and the dimensions of each fNIRS sample were $X_{HbO/HbR}\in {\mathbb{R}}^{36\times 50}$.

Because the original Shin et al. (2018) [49] dataset assigns an equal number of trials/blocks to each class and the same sliding-window configuration is applied to every block, the resulting samples are class-balanced for both tasks (1:1:1 for the n-back task and 1:1 for the WG task).

## 3. Method

**Figure 1:**
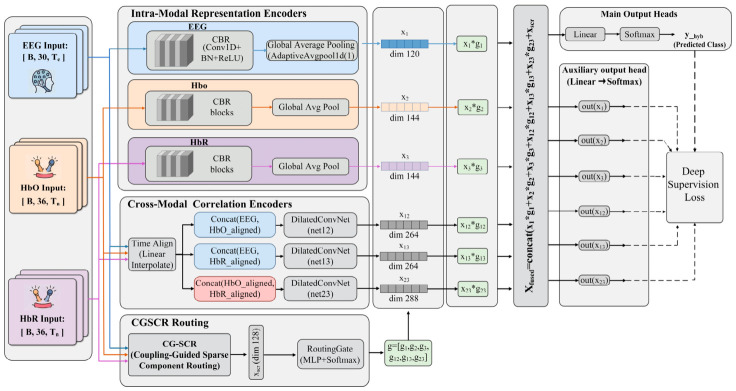
Overall architecture.

As illustrated in Figure 1, MGFNet is designed to address the temporal heterogeneity between EEG and fNIRS and to capture their cross-modal dependencies. The framework comprises three functional modules:

(1) **Intra-modal representation encoders**, which are designed to extract independent spatiotemporal features from each respective modality; (2) **cross-modal interaction encoders**, which temporally align paired modalities and use dilated convolutions to capture long-range cross-modal dependencies; and (3) **the CGSCR module**, which performs discrete sparse routing at the component level based on cross-modal coupling and subsequently generates soft branch-wise gating weights for adaptive fusion.

The gated branch-specific features are then fused and jointly optimized using a deep supervision strategy.

Overall, MGFNet follows a progressive fusion strategy. It first learns modality-specific representations from the EEG, HbO, and HbR streams, then models pairwise cross-modal interactions, and finally performs adaptive branch-wise fusion through the CGSCR module. The following subsections describe the intra-modal encoders, cross-modal interaction encoders, and the CGSCR module in detail.

### 3.1. Intra-Modal Representation Encoders

To preserve the modality-specific characteristics of heterogeneous physiological signals, MGFNet employs three parallel branches with independent (non-shared) parameters to encode the EEG, HbO, and HbR inputs, respectively. Each branch is built from stacked CBR modules, each consisting of a 1-D convolutional layer, batch normalization, and a ReLU activation. This design allows the network to learn modality-specific temporal patterns prior to cross-modal fusion.

Specifically, the EEG branch adopts six stacked CBR blocks with kernel sizes [9,3,3,9,3,3] and strides [4,1,1,4,1,1]. The relatively larger kernels and strides in the early layers help capture coarse global temporal patterns and reduce feature dimensionality, while the subsequent smaller convolutions refine local temporal details. In contrast, the HbO and HbR branches share the same architecture, with each branch consisting of six CBR blocks with kernel sizes of [5,3,3,5,3,3] and strides of [2,1,1,2,1,1]. Compared with EEG, the fNIRS branches use a gentler downsampling strategy to better match the lower temporal resolution of hemodynamic signals.

At the end of each branch, a global average pooling (GAP) layer is applied to obtain compact modality-specific feature vectors. Under the current network configuration, the EEG branch outputs the feature vector $x_{1}\in {\mathbb{R}}^{120}$, whereas the HbO and HbR branches output $x_{2},x_{3}\in {\mathbb{R}}^{144}$. These intra-modal representations provide the basis for the subsequent cross-modal interaction modeling and adaptive fusion.

Although these intra-modal branches preserve modality-specific information, hybrid BCI decoding also depends on the exchange of complementary information across modalities. Therefore, MGFNet further introduces dedicated cross-modal interaction encoders to explicitly model pairwise dependencies between EEG and fNIRS signals.

### 3.2. Cross-Modal Interaction Encoders

To capture complementary dependencies across modalities, MGFNet uses three pairwise cross-modal interaction encoders for EEG-HbO, EEG-HbR, and HbO-HbR.

Because EEG and fNIRS have different temporal resolutions, the fNIRS signals are first aligned to the EEG time axis through linear interpolation before pairwise interaction modeling. After temporal alignment, each modality pair is concatenated along the channel dimension and fed into a dedicated cross-modal encoder. Specifically, the three interaction branches produce the paired features $x_{12}$, $x_{13}$, and $x_{23}$, corresponding to EEG-HbO, EEG-HbR, and HbO-HbR interactions, respectively.

**Figure 2:**
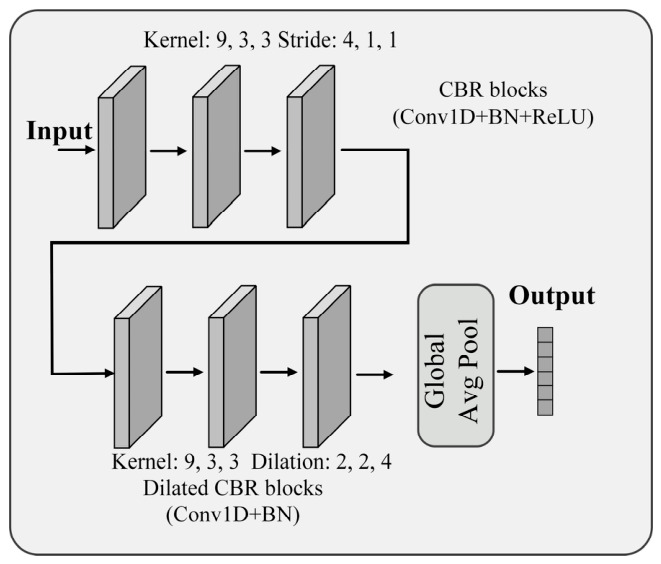
DilatedConvNet detail.

As illustrated in Figure 2, each cross-modal encoder is implemented as a DilatedConvNet. In the initial stage, the first convolutional layer employs a relatively large kernel size (k=9) and stride (s=4) to capture coarse global temporal patterns and reduce feature dimensionality. This is followed by two convolutional layers with smaller kernel sizes (k=3) and stride (s=1) to further refine local temporal information. After this local encoding stage, three dilated convolutional layers with dilation rates of [2,2,4] are introduced to enlarge the temporal receptive field and model longer-range dependencies between paired modalities. Finally, a Global Average Pooling (GAP) layer is applied to produce compact interaction features.

Under the current network configuration, the EEG-HbO and EEG-HbR branches output $x_{12},x_{13}\in {\mathbb{R}}^{264}$, while the HbO-HbR branch outputs $x_{23}\in {\mathbb{R}}^{288}$. Compared with static late fusion, these cross-modal interaction encoders allow the network to learn coordinated temporal patterns between paired modalities before the final adaptive fusion stage.

However, pairwise interaction features alone are insufficient for adaptive multimodal fusion because the relative reliability of different branches can vary across samples. To address this issue, we further introduce the CGSCR module, which estimates sample-specific cross-modal coupling and dynamically adjusts branch-wise fusion.

### 3.3. Coupling-Guided Sparse Component Routing (CGSCR)

**Figure 3:**
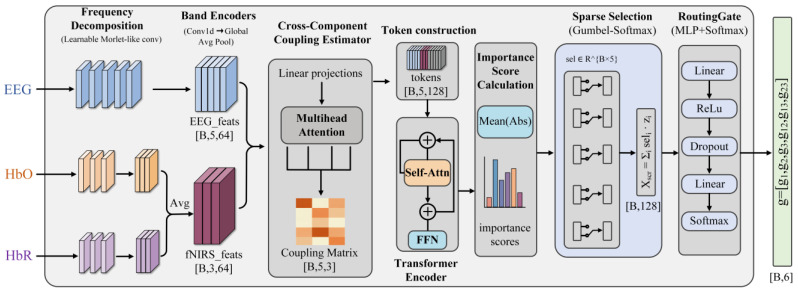
Detailed structure of the CGSCR module.

As illustrated in Figure 3, the proposed CGSCR module is designed to regulate feature fusion according to the current cross-modal coupling pattern. It operates through a two-stage mechanism: component-level discrete sparse routing and branch-level adaptive gating.

1. **Frequency Decomposition:** Given the EEG, HbO, and HbR inputs, CGSCR first performs multi-component decomposition using learnable Morlet-like convolutional filters. This operation decomposes EEG into multiple frequency bands and fNIRS into multiple scale components, providing a finer representation basis for subsequent cross-modal coupling analysis.
2. **Cross-Component Coupling Estimator:** A multi-head attention mechanism is used to compute the affinity matrix between EEG frequency bands and fNIRS components. This matrix quantifies which EEG oscillatory components are most strongly associated with hemodynamic variations in the current sample.

The decomposed components are then encoded by lightweight band encoders to obtain component-wise feature embeddings. Let the resulting EEG and fNIRS component features be $E\in {\mathbb{R}}^{B\times 5\times 64}$ and $F\in {\mathbb{R}}^{B\times 3\times 64}$, respectively. These features are projected into a shared 128-dimensional latent space, and a multi-head attention module is used to estimate sample-specific coupling between the modalities. The resulting coupling matrix $C\in {\mathbb{R}}^{B\times 5\times 3}$ characterizes how strongly each EEG component is associated with each fNIRS component in the current sample.

3. **Component-Level Sparse Routing and Branch-Level Gate Generation:** Based on the estimated coupling matrix, cross-modal tokens are constructed and fed into a two-layer Transformer encoder with eight attention heads, a hidden dimension of 128, a feedforward dimension of 512, and a dropout rate of 0.1. This encoder aggregates global contextual information across components and produces a latent representation z. The resulting token sequence is then converted into importance scores using the Mean (Abs) operation. To enable discrete yet differentiable routing at the component level, we employ a Gumbel-Softmax mechanism, whose temperature is initialized to 1.0 and learned jointly during training. This process yields a sparse selection vector, which is used to aggregate informative component tokens and obtain the routing feature $x_{scr}\in {\mathbb{R}}^{B\times 128}$. Finally, $x_{scr}$ is fed into a RoutingGate implemented as an MLP (128 → 128 → 6, dropout 0.1) followed by Softmax, producing a 6-dimensional branch-wise gating vector for the six intra-modal and cross-modal branches.

After branch-wise adaptive fusion is completed, the resulting hybrid representation is fed into the final prediction head. To stabilize training and improve the discriminability of intermediate branches, a deep supervision strategy is introduced, as described below.

### 3.4. Gated Fusion and Deep Supervision

After component-level sparse routing and branch-level adaptive gating, the gated branch features are concatenated with the routing feature to form the final hybrid representation, which is then fed into the main classifier for task prediction.

In addition to this final prediction head, auxiliary classifiers are attached to the three intra-modal branches and the three pairwise cross-modal branches, as indicated by the dashed connections in Figure 1. This deep supervision design encourages each branch to retain task-discriminative information during training rather than relying solely on the final fused representation.

Let $y_{hyb}$ denote the prediction of the final hybrid classifier, and let $y_{1}$, $y_{2}$, $y_{3}$, $y_{12}$, $y_{13}$, and $y_{23}$ denote the predictions of the EEG, HbO, HbR, EEG-HbO, EEG-HbR, and HbO-HbR branches, respectively. The overall training objective is defined as a weighted sum of the cross-entropy losses from these seven outputs:

(1) $$L=\lambda_{hyb}L_{CE}(y_{hyb},y)+\sum_{i\in \{1,2,3,12,13,23\}}\lambda_{i}L_{CE}(y_{i},y),$$

where L denotes the overall training objective loss of the MGFNet; $L_{CE}(\cdot ,\cdot )$ represents the standard cross-entropy loss function; y is the ground-truth label; $y_{hyb}$ denotes the prediction output from the final hybrid main classifier; $\lambda_{hyb}$ is the hyperparameter balancing the weight of the main classifier, set to 0.4; i∈{1,2,3,12,13,23} represents the index of the six auxiliary branches; $y_{i}$ represents the prediction output of the i auxiliary branch; and $\lambda_{i}$ is the hyperparameter controlling the weight of the i auxiliary branch, each set to 0.1. This objective jointly constrains the final fusion output and the intermediate branch representations, thereby stabilizing training and improving the consistency of feature learning across branches.

### 3.5. Evaluation Protocol and Implementation Details

MGFNet and its ablation variants were implemented in Python 3.10 with PyTorch (v2.5.1) and trained on a workstation equipped with an NVIDIA GeForce RTX 3090 GPU. To ensure fair comparisons with baseline models, standardized hyperparameter settings were used.

The Adam optimizer was selected with the learning rate set to $1\times 10^{-3}$ and the weight decay set to $1\times 10^{-3}$. The learning rate was kept constant throughout training and no learning-rate decay or warm-up scheduling was applied. According to the convergence characteristics of the two tasks, the number of training epochs was set to 60 for the n-back task and 70 for the WG task. The batch size was set to 64 for all experiments. To improve the training stability of the multi-stream network, we used a deep supervision loss. The weight of the main classifier was set to 0.4, while the weights for the six auxiliary branches (EEG, HbO, HbR, EEG-HbO, EEG-HbR, HbO-HbR) were each set to 0.1. This configuration balances global optimization with local feature learning.

To improve the transparency and reproducibility of the comparison, the available parameter settings of the compared methods are summarized in Table 1. The settings of the external comparison methods were compiled from the corresponding original publications, while the settings of MGFNet correspond to the implementation used in the present study.

Following recent physiological-signal classification studies [53,54], we evaluated model performance using four complementary metrics: Accuracy, Precision, Recall, and F1-score. Together, these metrics characterize overall classification correctness, false-positive control, sensitivity to the target class, and the balance between precision and recall. These metrics are computed from the standard confusion-matrix entries: true positives (TP), true negatives (TN), false positives (FP), and false negatives (FN).

Accuracy: Measures the overall correct classification rate of the model on the test set.

(2) $$Accuracy=\frac{TP+TN}{TP+TN+FP+FN},$$

Precision: Represents the proportion of true positive predictions among all positive predictions made by the model, indicating its ability to avoid false alarms.

(3) $$Precision=\frac{TP}{TP+FP},$$

Recall: Represents the proportion of actual positive samples correctly identified by the model, reflecting its sensitivity to the target class.

(4) $$Recall=\frac{TP}{TP+FN},$$

F1-score: Serving as the harmonic mean of Precision and Recall, this metric is used to assess the comprehensive performance of the model, particularly under conditions of class imbalance.

(5) $$F1-score=2\times \frac{Precision\times Recall}{Precision+Recall},$$

All reported results in this paper correspond to held-out test performance under a within-subject evaluation protocol. For each subject, the original trials/task blocks were randomly divided into 80% for training and 20% for held-out testing. To prevent information leakage caused by overlapping sliding windows, this partitioning was performed before sliding-window segmentation, and windows were subsequently generated separately within each subset. Therefore, no windows originating from the same original trial/block were shared across the training and test sets.

A five-fold cross-validation procedure was applied only within the 80% training subset for validation and model selection [55]. Importantly, the fold assignment was conducted at the trial/block level rather than at the window level, such that all windows derived from the same original trial/block remained in the same fold. Thus, the final reported results were obtained from unseen test trials/blocks rather than from the validation folds. When results are reported as Mean ± Std, the statistics are computed across subjects.

In addition to the above classification metrics, the information transfer rate (ITR) was calculated as an auxiliary measure to provide a practical interpretation of the decoding efficiency of different methods. The amount of information transmitted per decision was computed as:

(6) $$B=\log_{2}(N)+P\log_{2}(P)+(1-P)\log_{2}\left(\frac{1-P}{N-1}\right),$$

where B denotes bits/trial, N is the number of target classes, and B is the classification accuracy expressed as a decimal value. The ITR in bits/min was further obtained by:

(7) $$\mathrm{ITR}=\frac{B\times 60}{T},$$

where T is the decision time. In this study, T was set to 5 s, corresponding to the sliding-window length used for sample construction. Accordingly, N was set to 3 for the n-back task and 2 for the WG task. The ITR values in Table 2 and Table 3 were calculated based on the reported accuracy of each method under this unified setting.

## 4. Results

### 4.1. Performance Comparison and ITR Evaluation

All experiments in this section followed the leakage-free within-subject evaluation protocol described in Section 3.5 unless otherwise specified. Under this protocol, MGFNet was compared with representative literature-reported methods, including traditional machine-learning methods, deep neural networks, and multimodal fusion approaches, on the n-back task (three-class classification) and the WG task (binary classification). To improve the transparency of the comparison, the available parameter settings of the com-pared methods are summarized in Table 1. For MGFNet, the results reported in Table 2 and Table 3 denote the mean subject-wise held-out test performance across all 26 subjects. In addition to accuracy, the information transfer rate (ITR) was calculated for all compared methods according to the unified decision-time setting described in Section 3.5.

Table 2 summarizes the comparison results for the n-back task. Among the literature-reported methods, MBC-ATT achieved the highest accuracy of 98.13%. Under the present within-subject held-out protocol, MGFNet reached a mean accuracy of 99.40%, corresponding to improvements of 1.27 percentage points over MBC-ATT and 4.30 percentage points over STFT-MDNF. In terms of ITR, MGFNet obtained 18.31 bits/min, exceeding the MBC-ATT value of 17.19 bits/min. For descriptive context, the subject-wise accuracies reported by MBC-ATT were 98.13 ± 4.20% (range: 81.82–99.99%), whereas MGFNet reached 99.40 ± 0.61% (range: 97.95–99.99%) in the present study. Since these results were obtained from different studies rather than from a unified reimplementation, they are presented as descriptive comparisons under a comparable evaluation setting.

Table 3 presents the comparison results for the WG task. Among the literature-reported methods, MBC-ATT achieved the highest reported accuracy of 98.61%, followed by EF-Net [56] with 96.29%. Under the present within-subject held-out evaluation protocol, MGFNet reached a mean accuracy of 99.03%, corresponding to a 0.42 percentage-point improvement over the literature-reported MBC-ATT result and a 2.74 percentage-point improvement over EF-Net. MGFNet also achieved the highest ITR on this task, reaching 11.05 bits/min compared with 10.73 bits/min for MBC-ATT. The subject-wise accuracies reported in the MBC-ATT study correspond to 98.61 ± 1.47% with a range of 94.44–99.99%, whereas MGFNet achieved 99.03 ± 1.16% with a range of 95.83–99.99% in the present study. These values provide a descriptive comparison under a comparable protocol.

Overall, MGFNet achieved mean held-out test accuracies of 99.40% and 99.03% on the n-back and WG tasks, respectively, and obtained the highest ITR values among the compared methods. These results indicate that the proposed fusion and representation learning strategy is effective under the present within-subject offline evaluation setting.

**Figure 4:**
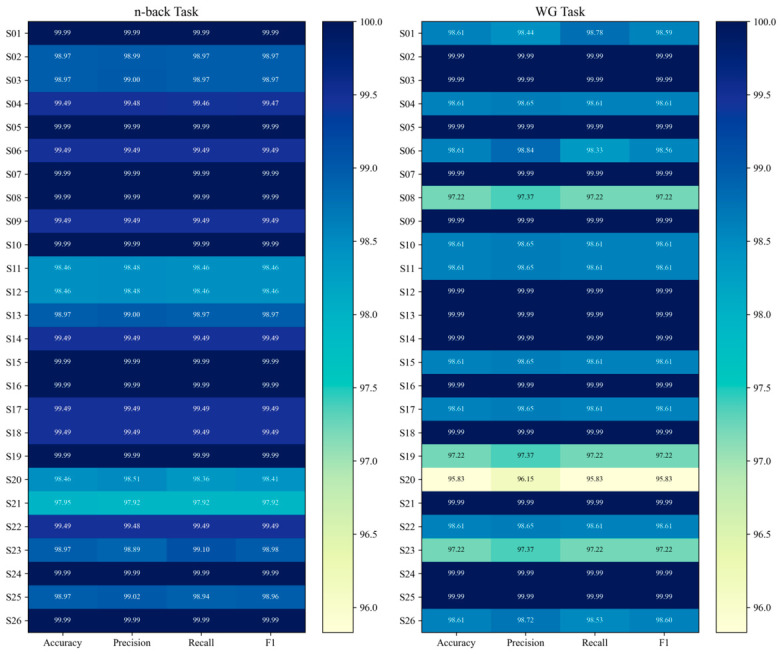
Subject-wise performance heatmaps of the complete MGFNet model across all 26 subjects. The left panel shows the n-back task (three-class classification), and the right panel shows the WG task (binary classification). The four evaluation metrics are Accuracy, Precision, Recall, and F1-score. Color intensity represents the performance level in percentage, and exact metric values are displayed within each cell.

To further assess model stability at the individual level, we plotted subject-specific performance heatmaps based on the held-out test results of all 26 subjects. As shown in Figure 4, MGFNet achieved consistently high values across Accuracy, Precision, Recall, and F1-score for most subjects in both tasks. This indicates that the overall performance was not driven by a small number of high-performing participants but instead reflected stable performance across the participant pool. Moreover, the consistently high subject-wise metrics suggest that MGFNet can adapt to subject-specific neural patterns despite inter-individual variability. These observations are also consistent with the low across-subject standard deviations reported above, further supporting the stability of MGFNet under the present within-subject protocol.

### 4.2. Ablation Study

To evaluate the contributions of individual MGFNet modules, ablation studies were conducted on both the n-back task (three-class classification) and the WG task (binary classification). All ablation experiments followed the same data processing pipeline and the leakage-free within-subject evaluation protocol described in Section 2.2 and Section 3.5, respectively. Unless otherwise specified, the reported results correspond to subject-wise held-out test performance and are summarized as mean ± standard deviation across subjects. We investigated three components of the architecture: (1) the contribution of the CGSCR module to adaptive fusion; (2) the contribution of the Cross-Modal Interaction Encoders; and (3) the impact of the Intra-Modal Representation Encoders on performance and stability.

Table 4 summarizes the ablation results on the n-back and WG tasks. On the n-back task, the full MGFNet achieved a mean held-out test accuracy of 99.40 ± 0.61% across subjects. Replacing the CGSCR module with a static feature-concatenation strategy reduced the accuracy to 98.78 ± 1.19%, indicating that static fusion is less effective in exploiting cross-modal complementarity. Removing the cross-modal interaction encoders produced the largest performance drop (accuracy decreased to 98.28 ± 1.66%), whereas removing the intra-modal representation encoders produced a smaller drop (98.85 ± 0.82%). Overall, the magnitude of performance degradation followed the order of *w*/*o* Cross-Modal, *w*/*o* CGSCR, and *w*/*o* Intra-Modal. These results suggest that explicit cross-modal interaction modeling plays a central role in cognitive workload discrimination, while intra-modal representation learning and CGSCR-based adaptive fusion further improve the stability of the learned features.

On the WG task, the full MGFNet achieved a mean held-out test accuracy of 99.03 ± 1.16% across subjects. Similar to the n-back task, replacing CGSCR-based adaptive fusion with static fusion reduced the accuracy to 98.29 ± 1.26%, and removing the intra-modal representation encoders reduced the accuracy to 98.45 ± 1.59%. Removing the cross-modal interaction encoders again caused the largest decrease, reducing the accuracy to 97.54 ± 2.55%. In particular, the *w*/*o* Cross-Modal setting decreased accuracy by 1.49 percentage points relative to the full model, suggesting that cross-modal dependency modeling is also important for robust discrimination in the WG task.

To assess whether the observed performance differences were statistically significant, two-sided paired *t*-tests were applied to the subject-wise held-out test accuracies (N = 26, df = 25). This test was selected because the full MGFNet and each ablation variant were evaluated on the same set of subjects under the same evaluation protocol, such that the resulting accuracies formed paired observations rather than independent samples. Therefore, the paired *t*-test is suitable for assessing whether the mean within-subject performance difference between two matched model settings differs significantly from zero while accounting for inter-subject variability.

On the n-back task (Table 5), the full MGFNet significantly outperformed *w*/*o* Cross-Modal (*t* = 3.809, *p* = 0.0008), *w*/*o* Intra-Modal (*t* = 2.802, *p* = 0.0097), and *w*/*o* CGSCR (*t* = 2.091, *p* = 0.0469), with all comparisons reaching statistical significance (*p* < 0.05). These findings further indicate that the cross-modal interaction encoders, intra-modal representation encoders, and CGSCR module each contribute to the final performance, with the cross-modal interaction encoders showing the largest contribution.

The same paired *t*-test analysis was performed for the WG task. The improvements of the full MGFNet over *w*/*o* Cross-Modal (*t* = 2.531, *p* = 0.0180), *w*/*o* Intra-Modal (*t* = 2.102, *p* = 0.0458), and *w*/*o* CGSCR (*t* = 2.209, *p* = 0.0366) were all statistically significant (*p* < 0.05). These results further support the effectiveness of the proposed intra-modal and cross-modal encoders, together with the CGSCR module, in improving the discriminability and stability of the learned representations.

To evaluate the contribution of each modality to cognitive-state discrimination, modality ablation experiments were conducted under three input settings: EEG-only, fNIRS-only, and EEG + fNIRS. All other data processing steps and evaluation settings were identical to those described above, and the reported modality-ablation results were based on subject-wise held-out test performance.

**Figure 5:**
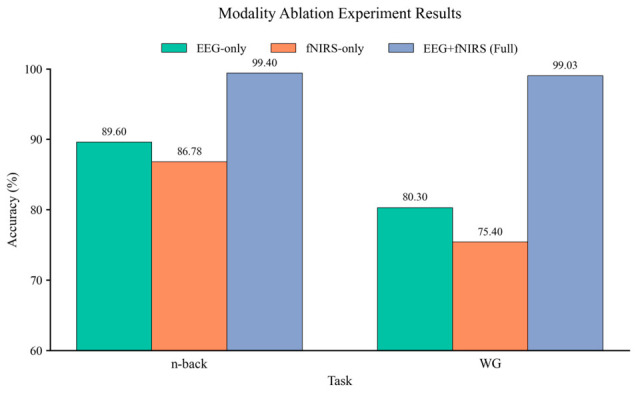
Modality ablation results on the n-back and WG tasks under EEG-only, fNIRS-only, and EEG + fNIRS settings.

As illustrated in Figure 5, the EEG-only setting achieved higher performance than the fNIRS-only setting in both tasks, while the multimodal EEG + fNIRS setting achieved the best overall performance. These findings support the complementary nature of EEG and fNIRS signals and indicate that multimodal fusion is beneficial for improving decoding performance.

### 4.3. Noise Analysis and Robustness Evaluation

The robustness analysis was deliberately focused on the EEG modality. This choice was motivated by practical considerations in physiological-signal acquisition: compared with fNIRS, EEG is generally more susceptible to electrophysiological and motion-related artifacts such as EOG and EMG contamination. Perturbing only the EEG stream therefore provides a controlled and practically meaningful setting for evaluating whether the proposed CGSCR-based fusion mechanism can adapt to asymmetric modality degradation.

The robustness analysis consisted of two complementary experiments. First, we adopted a clean-training/noisy-testing protocol to isolate the inference-time response of the fusion architecture under EEG degradation. Second, we further conducted a noise-aware training experiment to examine whether the advantage of MGFNet persists when both architectures are explicitly exposed to noisy EEG during optimization.

In the clean-training/noisy-testing protocol, both the full MGFNet and the static-fusion variant without CGSCR were trained on clean EEG-fNIRS data. During testing, additive white Gaussian noise (AWGN) was injected only into the EEG signals of the held-out test subset, while the temporally paired fNIRS signals were kept unchanged. This design ensured identical training conditions for the two models, allowing performance differences under test-time corruption to be attributed to the architectural difference between CGSCR-based adaptive fusion and static fusion. The contaminated EEG signal, denoted as ${\hat{X}}_{EEG}$, is defined as follows:

(8) $${\hat{X}}_{EEG}=X_{EEG}+N(0,\sigma_{noise}^{2}),$$

where the noise variance $\sigma_{noise}^{2}$ is determined by the target Signal-to-Noise Ratio (SNR) and the original signal power $P_{signal}$:

(9) $$\sigma_{noise}^{2}=\frac{P_{signal}}{10^{\frac{SNR}{10}}},$$

where ${\hat{X}}_{EEG}$ represents the corrupted EEG signal after noise injection; $X_{EEG}$ denotes the original, clean EEG signal; $N(0,\sigma_{noise}^{2})$ denotes the AWGN following a normal distribution with a mean of 0 and a variance of $\sigma_{noise}^{2}$; $\sigma_{noise}^{2}$ is the power (variance) of the injected noise; $P_{signal}$ represents the original signal power (variance) of the clean EEG signal $X_{EEG}$; and SNR is the target Signal-to-Noise Ratio expressed in decibels (dB), which controls the intensity of the injected noise.

**Figure 6:**
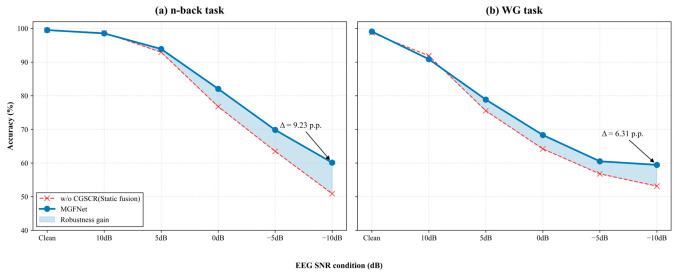
Robustness evaluation under test-time EEG noise injection for the (**a**) n-back task and (**b**) WG task. Classification accuracies of MGFNet and the static-fusion variant without CGSCR are shown under different EEG SNR conditions. AWGN was injected into the EEG modality only, while the paired fNIRS signals were kept unchanged.

We evaluated model performance under five EEG noise levels (10 dB, 5 dB, 0 dB, −5 dB, and −10 dB), together with the clean condition (Figure 6). These settings span a broad range of perturbation intensities, from mild interference to noise-dominant conditions. In particular, 0 dB indicates equal signal and noise power, whereas negative SNR values correspond to progressively more severe corruption.

For the n-back task, MGFNet and the static-fusion variant showed comparable performance under the clean and 10 dB conditions. As the SNR decreased, the advantage of MGFNet became more evident. Specifically, MGFNet outperformed the static-fusion variant by 0.95, 5.28, 6.35, and 9.23 percentage points at 5, 0, −5, and −10 dB, respectively. At −10 dB, MGFNet achieved an accuracy of 60.12%, whereas the static-fusion variant dropped to 50.89%.

A similar trend was observed for the WG task. Although the static-fusion variant performed slightly better at 10 dB, MGFNet achieved higher accuracy under stronger EEG corruption, with gains of 3.32, 4.11, 3.74, and 6.31 percentage points at 5, 0, −5, and −10 dB, respectively. At −10 dB, MGFNet maintained an accuracy of 59.46%, compared with 53.15% for the static-fusion variant. These results indicate that CGSCR-based adaptive fusion is more effective than static fusion under controlled test-time EEG degradation.

To examine whether this advantage persisted when noisy patterns were incorporated during optimization, we conducted a 2 × 2 factorial experiment with two architectural settings (MGFNet and the static-fusion variant) and two training regimes (clean training and noise-aware training). During noise-aware training, each EEG training sample was independently corrupted with a probability of 0.8. The corresponding SNR was randomly selected from {10, 5, 0, −5, −10} dB, while the remaining 20% of training samples were kept clean. The validation set was corrupted using the same strategy, and the fNIRS modality remained unchanged throughout training and evaluation. All four configurations were trained from scratch using the same optimizer, random seed, batch size, and number of epochs.

**Figure 7:**
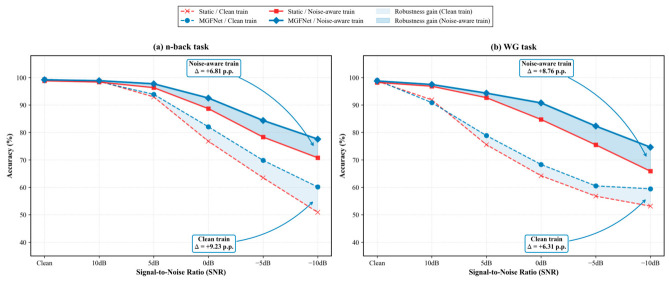
Effect of noise-aware training on robustness against EEG corruption for the (**a**) n-back task and (**b**) WG task. Four configurations are compared: static fusion with clean training, static fusion with noise-aware training, MGFNet with clean training, and MGFNet with noise-aware training. Dashed curves denote clean training, while solid curves denote noise-aware training.

Figure 7 presents the results of the noise-aware training experiment. Compared with clean training, noise-aware training improved robustness for both architectures under severe EEG corruption. At −10 dB on the n-back task, the accuracy increased from 50.89% to 70.77% for the static-fusion variant and from 60.12% to 77.58% for MGFNet. On the WG task, the corresponding improvements were +12.72 percentage points for the static-fusion variant and +15.17 percentage points for MGFNet. These results indicate that exposure to noisy EEG during training improves robustness regardless of the fusion architecture.

Importantly, under matched noise-aware training, MGFNet still achieved higher accuracy than the static-fusion variant at moderate-to-severe corruption levels. At −10 dB, MGFNet exceeded the static-fusion variant by 6.81 percentage points on the n-back task and 8.76 percentage points on the WG task. Similar advantages were observed at −5 dB and 0 dB. These findings indicate that the advantage of MGFNet is not merely caused by the clean-training/noisy-testing protocol, but is preserved when both models are trained with noisy EEG exposure.

Under clean-condition testing, noise-aware training introduced only a minor accuracy reduction for MGFNet, with decreases of 0.23 percentage points on the n-back task and 0.27 percentage points on the WG task. These reductions were smaller than those observed for the static-fusion variant. At −10 dB, MGFNet also retained the highest proportion of its clean-condition performance, suggesting a favorable robustness-accuracy trade-off.

Taken together, the two robustness analyses provide complementary evidence. The clean-training/noisy-testing experiment isolates the architectural contribution of the CGSCR module under fixed training conditions, while the noise-aware training experiment shows that this advantage persists when both architectures are exposed to noisy EEG during optimization. These results suggest that CGSCR-based adaptive fusion improves resilience to controlled single-modality degradation under both clean-training and noise-aware training protocols.

### 4.4. Visualization of Learned Representations

To evaluate the learned feature representations, t-distributed stochastic neighbor embedding (t-SNE) was used to project high-dimensional feature vectors into a two-dimensional space. We compared the feature distributions across the following three stages:

(1) Raw Data: The preprocessed and time-aligned EEG and fNIRS (HbO/HbR) signals were concatenated at the window level, flattened into high-dimensional vectors after z-score normalization, and subsequently projected. This illustrates the inherent degree of inter-class overlap within the input feature space.

(2) Static Fusion: The CGSCR module was removed and replaced with standard multimodal feature concatenation as a static fusion strategy. The fused feature vectors immediately preceding the classification head were extracted and projected. This stage demonstrates the baseline inter-class separability and feature distribution achieved by the network in the absence of dynamic feature selection.

(3) MGFNet: The fused feature representations generated by the complete MGFNet were extracted and projected. This stage was used to examine the effect of the CGSCR module on intra-class compactness and inter-class separability.

To ensure reproducibility, all t-SNE visualizations were generated using identical hyperparameter settings, including perplexity = 30, number of iterations = 1000, and a fixed random seed. Visualizations were generated from the held-out test samples of both the n-back and WG tasks.

**Figure 8:**
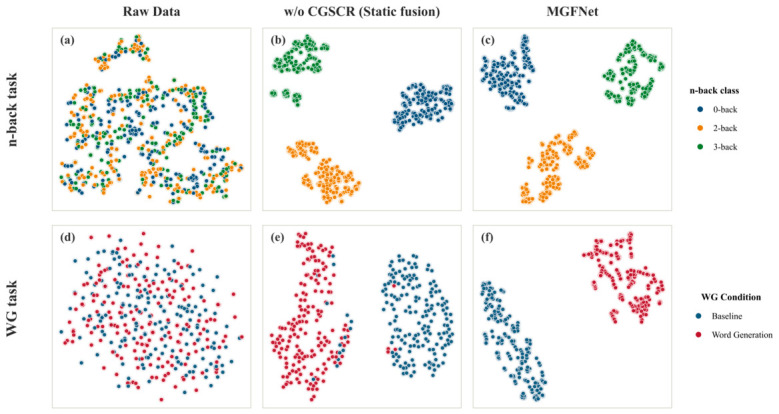
t-SNE visualization of feature representations on the n-back and WG tasks. The top row shows the n-back task results: (**a**) raw data distribution, (**b**) static fusion model without CGSCR, and (**c**) MGFNet. The bottom row shows the corresponding WG task results: (**d**) raw data distribution, (**e**) static fusion model without CGSCR, and (**f**) MGFNet. MGFNet shows improved class separability and more compact clustering compared with the raw input space and the static-fusion variant.

As shown in Figure 8a, the raw data distribution for the n-back task exhibited substantial class overlap, with samples from different cognitive workload levels intertwined in the two-dimensional space. This indicates that the raw input features do not form a clear discriminative structure. In contrast, the static-fusion model in Figure 8b formed more distinguishable clusters among the 0-back, 2-back, and 3-back classes, which is consistent with its strong classification performance.

As shown in Figure 8c, MGFNet further increased intra-class compactness while preserving inter-class separability compared with the static-fusion model. The tighter clusters suggest that CGSCR-based adaptive routing and branch-wise gating reduce task-irrelevant intra-class variation and improve the discriminability of the learned representations. This observation is consistent with the lower performance variance reported in the ablation results.

A similar trend was observed in the WG task. The raw data distribution in Figure 8d showed no clear separable structure, whereas both the static-fusion model and MGFNet achieved evident binary separation, as shown in Figure 8e,f. Compared with the static-fusion model, MGFNet exhibited tighter intra-class distributions and smaller inter-class overlap, suggesting improved discriminative robustness for boundary samples.

### 4.5. Confusion Matrix Analysis

**Figure 9:**
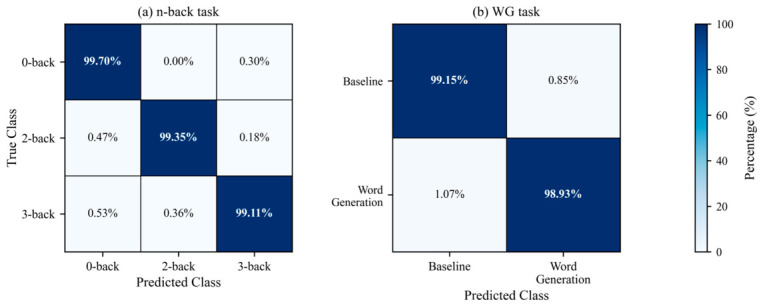
Global confusion matrices aggregated across all 26 subjects for (**a**) the n-back task and (**b**) the WG task. The matrices were obtained by pooling sample-level predictions from the held-out test subsets of all participants and were row-normalized by the true class, with all entries expressed as percentages.

To further examine class-wise prediction behavior, we constructed global confusion matrices by pooling sample-level predictions from the held-out test subsets of all 26 subjects, as shown in Figure 9.

For the n-back task, the global confusion matrix shows strong class-wise discrimination among the three workload levels, with recall rates of 99.70%, 99.35%, and 99.11% for 0-back, 2-back, and 3-back, respectively. Off-diagonal errors remained low, with the largest confusion observed for 3-back → 0-back (0.53%), 2-back → 0-back (0.47%), and 3-back → 2-back (0.36%). For the WG task, MGFNet achieved recall values of 99.15% for the Baseline condition and 98.93% for the WG condition, with 0.85% of Baseline samples misclassified as WG and 1.07% of WG samples misclassified as Baseline. These results indicate that MGFNet maintains stable class-wise prediction performance across different cognitive task conditions.

## 5. Discussion

### 5.1. Overall Effectiveness of MGFNet

The experimental results demonstrate that MGFNet achieved strong decoding performance on both the n-back and WG tasks under the leakage-free within-subject held-out evaluation protocol. Compared with representative literature-reported methods, MGFNet achieved higher mean accuracies and ITR values on both tasks. These findings suggest that the proposed multi-granularity fusion framework is effective for hybrid EEG-fNIRS decoding under the present offline evaluation setting.

The subject-wise heatmaps indicate that the overall performance was broadly shared across participants rather than driven by a small group of high-performing individuals. In addition, the global confusion matrices showed stable class-wise prediction behavior for both tasks. These results suggest that MGFNet can learn discriminative representations across different cognitive task conditions while maintaining stable within-subject decoding performance.

### 5.2. Contribution of Multi-Granularity Fusion and CGSCR

The ablation results clarify the contribution of each component in MGFNet. Removing the cross-modal interaction encoders produced the largest performance drop on both tasks, indicating that explicit modeling of EEG-fNIRS dependencies plays a central role in cognitive-state discrimination. This result is consistent with the motivation of the proposed framework: although EEG and fNIRS provide complementary temporal and hemodynamic information, their relationship is not fully captured by simple feature concatenation.

The CGSCR module further contributes to adaptive multimodal fusion. Compared with the static-fusion variant, the full MGFNet achieved higher accuracy and lower across-subject variability. This suggests that sample-specific routing and branch-wise gating are beneficial for selecting informative feature components according to the current cross-modal coupling pattern. Unlike static fusion, which combines branch features with fixed weights, CGSCR-based fusion adjusts the contribution of each branch according to the current input sample.

The visualization results also support this interpretation. In the t-SNE projections, the complete MGFNet produced more compact intra-class distributions and clearer inter-class separation than the raw input space and the static-fusion variant. This finding suggests that the combination of intra-modal representation learning, cross-modal interaction modeling, and CGSCR-based adaptive fusion improves the discriminability of the learned feature space.

### 5.3. Robustness Under EEG Degradation

The robustness experiments characterize the role of CGSCR-based adaptive fusion under modality degradation. In the clean-training/noisy-testing setting, the performance gap between MGFNet and the static-fusion variant widened as EEG corruption increased. This indicates that the proposed adaptive fusion mechanism can better tolerate asymmetric modality degradation when the EEG stream becomes unreliable.

To address the concern that a clean-training/noisy-testing protocol alone may not reflect noise-aware training, we additionally examined a noise-aware-training setting. The results showed that noise-aware training improved robustness for both MGFNet and the static-fusion variant. MGFNet still achieved higher accuracy than the static-fusion variant under matched noise-aware training, especially at moderate-to-severe EEG corruption levels. Therefore, the robustness advantage of MGFNet was not merely caused by the clean-training/noisy-testing protocol but was preserved when both models were exposed to noisy EEG during optimization.

The robustness analysis was designed as a controlled, mechanism-oriented evaluation. AWGN provides a reproducible perturbation setting for examining the response of the fusion architecture to single-modality degradation. However, it does not fully represent all real-world EEG artifacts, such as EOG, EMG, electrode displacement, and motion-related noise. Future studies should evaluate the proposed framework under more realistic artifact conditions and online acquisition scenarios.

### 5.4. Practical Implications and Comparison with Existing Methods

Beyond classification accuracy, ITR provides a practical measure of decoding efficiency from a BCI perspective. Under the unified 5 s decision window used in this study, MGFNet achieved the highest ITR values among the compared methods on both the n-back and WG tasks. This indicates that the performance gain of MGFNet was reflected not only in accuracy but also in information-transfer efficiency under the present offline evaluation setting.

At the same time, comparisons with existing methods should be interpreted with caution. Several baseline results were collected from previous studies rather than reimplemented under an identical experimental environment. Therefore, the comparison with MBC-ATT and other external methods should be regarded as a literature-based descriptive comparison under matched evaluation settings rather than a strict cross-study statistical ranking. To improve transparency, the available parameter settings of the compared methods were summarized in the Section 3, and the comparison with MBC-ATT was further supplemented with descriptive statistics derived from the subject-wise results reported in the original study.

Despite these limitations, the consistent improvements observed in the overall performance comparison, ablation study, robustness analysis, t-SNE visualization, and confusion-matrix analysis jointly support the effectiveness of the proposed architecture under the present within-subject offline evaluation protocol.

### 5.5. Limitations and Future Work

Although MGFNet achieved promising results, several limitations should be acknowledged. The current experiments were conducted under a within-subject offline evaluation protocol, which is suitable for validating the proposed architecture but does not fully reflect cross-subject, cross-session, or online BCI scenarios. In addition, the robustness analysis used AWGN to simulate controlled EEG degradation. This setting helps isolate the effect of asymmetric modality corruption, but it cannot cover the full complexity of real artifacts such as EOG, EMG, motion noise, electrode instability, or fNIRS optode coupling changes. Future work will further evaluate MGFNet under cross-subject, cross-session, and online BCI settings, and will incorporate more realistic artifact-contaminated recordings to assess its robustness in practical scenarios.

## 6. Conclusions

In this study, we proposed MGFNet, a multi-granularity fusion network for hybrid EEG-fNIRS decoding. The framework integrates intra-modal representation learning, cross-modal interaction modeling, and the CGSCR module to capture both modality-specific characteristics and latent cross-modal dependencies. By introducing CGSCR-based adaptive fusion, MGFNet provides a sample-specific mechanism for integrating heterogeneous electrophysiological and hemodynamic information.

Experiments on a public EEG-fNIRS benchmark demonstrated that MGFNet achieved accuracies of 99.40% and 99.03% on the n-back and WG tasks, respectively, under a leakage-free within-subject held-out evaluation protocol. MGFNet also obtained the highest ITR values among the compared methods. Ablation studies confirmed the contributions of the intra-modal encoders, the cross-modal interaction encoders, and the CGSCR module. Robustness experiments further showed that MGFNet maintained higher performance than a static-fusion variant under controlled EEG degradation. These results support the effectiveness of multi-granularity interaction modeling and CGSCR-based adaptive fusion for hybrid BCI decoding.

## Figures and Tables

**Table 1:** Summary of available parameter settings for the compared methods. The settings of external comparison methods were summarized from the corresponding original publications when available.

Method	Input/Feature	Model/Fusion	Key Settings
SVM	ERD/ERS + ΔHbO + ΔHbR	SVM classifier	Window = 3 s; Feature dimension = 102
DNN	ERD/ERS + ΔHbO + ΔHbR	Fully connected DNN	Window = 3 s; hidden layers = 4; neurons/layer = 60; activation = ELU
CNN-LSTM	Recurrence-plot images	Time-distributed CNN-LSTM	RP window = 5 s; ε = 0.1; LSTM cells = 512; CV = 10-fold
STFT-MDNF	STFT EEG images + fNIRS spectral entropy	DenseNet201-based fusion	STFT = 1 s/0.5 s overlap; epochs = 50; batch size = 8; lr = $1\times 10^{-4}$
EF-Net	EEG = 500 × 30; fNIRS = 25 × 72	Two-branch CNN fusion	Dropout = 0.5; epochs = 70; batch size = 32; lr = $1\times 10^{-3}$
MBC-ATT	EEG + fNIRS	CNN branches + 4-head cross-modal attention	Adam; lr = $1\times 10^{-3}$; epochs = 60/70
MGFNet	EEG + HbO + HbR	Intra-modal + cross-modal + CGSCR	Adam; batch size = 64; lr = $1\times 10^{-3}$; epochs = 60/70

**Table 2:** Accuracy and Information Transfer Rate (ITR) comparison of different methods on the n-back task. Baseline accuracies are quoted from the MBC-ATT study and corresponding prior publications under comparable within-subject evaluation settings. ITR values were calculated using N = 3 classes and T = 5 s. Bold indicates the proposed method and the best results.

Method	Accuracy (%)	ITR (bits/min)
SVM	83.00	9.09
DNN	87.00	10.77
CNN-LSTM	88.41	11.42
STFT-MDNF	95.10	15.05
MBC-ATT	98.13 ± 4.20	17.19
**MGFNet (Ours)**	**99.40 ± 0.61**	**18.31**

**Table 3:** Accuracy and Information Transfer Rate (ITR) comparison of different methods on the WG task. Baseline accuracies are quoted from the MBC-ATT study and corresponding prior publications under comparable within-subject evaluation settings. ITR values were calculated using N = 2 classes and T = 5 s. Bold indicates the proposed method and the best results.

Method	Accuracy (%)	ITR (bits/min)
SVM	73.99	2.08
DNN	92.00	7.17
STFT-MDNF	93.10	7.65
EF-Net	96.29	9.25
MBC-ATT	98.61 ± 1.47	10.73
**MGFNet (Ours)**	**99.03 ± 1.16**	**11.05**

**Table 4:** Performance comparison of ablation models on n-back and WG tasks (%).

Model Variant	n-Back Acc (%)	WG Acc (%)
**MGFNet (Full)**	**99.40 ± 0.61**	**99.03 ± 1.16**
*w*/*o* Cross-Modal	98.28 ± 1.66	97.54 ± 2.55
*w*/*o* Intra-Modal	98.85 ± 0.82	98.45 ± 1.59
*w*/*o* CGSCR (static fusion)	98.78 ± 1.19	98.29 ± 1.26

Note: Bold indicates the proposed method and the best results.

**Table 5:** Statistical significance analysis based on subject-wise held-out test accuracies (paired *t*-test).

Task	Comparison	t-Value	*p*-Value	Significance
n-back	Full vs. *w*/*o* Cross-Modal	3.809	0.0008	***
	Full vs. *w*/*o* Intra-Modal	2.802	0.0097	**
	Full vs. *w*/*o* CGSCR	2.091	0.0469	*
WG	Full vs. *w*/*o* Cross-Modal	2.531	0.0180	*
	Full vs. *w*/*o* Intra-Modal	2.102	0.0458	*
	Full vs. *w*/*o* CGSCR	2.209	0.0366	*

Note: * *p* < 0.05, ** *p* < 0.01, *** *p* < 0.001.

## Data Availability

The dataset analyzed during the current study is publicly available at https://doc.ml.tu-berlin.de/simultaneous_EEG_NIRS/ (accessed on 14 October 2025).
